# Absence of Ret Signaling in Mice Causes Progressive and Late Degeneration of the Nigrostriatal System

**DOI:** 10.1371/journal.pbio.0050039

**Published:** 2007-02-13

**Authors:** Edgar R Kramer, Liviu Aron, Geert M. J Ramakers, Sabine Seitz, Xiaoxi Zhuang, Klaus Beyer, Marten P Smidt, Rüdiger Klein

**Affiliations:** 1 Department of Molecular Neurobiology, Max-Planck Institute of Neurobiology, Martinsried, Germany; 2 Department of Pharmacology and Anatomy, Rudolf Magnus Institute of Neuroscience, University Medical Center Utrecht, Utrecht, The Netherlands; 3 Department of Neuroimmunology, Max Planck Institute of Neurobiology, Martinsried, Germany; 4 Institute for Clinical Neuroimmunology, Ludwig Maximilians University, Munich, Germany; 5 Department of Neurobiology, Pharmacology and Physiology, University of Chicago, Chicago, Illinois, United States of America; 6 Department of Metabolic Biochemistry, Adolf Butenandt Institute, Munich, Germany; University of Edinburgh, United Kingdom

## Abstract

Support of ageing neurons by endogenous neurotrophic factors such as glial cell line–derived neurotrophic factor (GDNF) and brain-derived neurotrophic factor (BDNF) may determine whether the neurons resist or succumb to neurodegeneration. GDNF has been tested in clinical trials for the treatment of Parkinson disease (PD), a common neurodegenerative disorder characterized by the loss of midbrain dopaminergic (DA) neurons. BDNF modulates nigrostriatal functions and rescues DA neurons in PD animal models. The physiological roles of GDNF and BDNF signaling in the adult nigrostriatal DA system are unknown. We generated mice with regionally selective ablations of the genes encoding the receptors for GDNF *(Ret)* and BDNF *(TrkB)*. We find that *Ret,* but not *TrkB,* ablation causes progressive and adult-onset loss of DA neurons specifically in the substantia nigra pars compacta, degeneration of DA nerve terminals in striatum, and pronounced glial activation. These findings establish Ret as a critical regulator of long-term maintenance of the nigrostriatal DA system and suggest conditional Ret mutants as useful tools for gaining insights into the molecular mechanisms involved in the development of PD.

## Author Summary

What does a neuron need to survive? Our body produces its own survival factors for neurons, so-called neurotrophic factors, which have additional roles in neuron differentiation, growth, and function. Declining production of a neurotrophic factor or impaired signal transduction in ageing neurons may contribute to pathological neurodegeneration in humans. Glial cell line–derived neurotrophic factor (GDNF) and brain-derived neurotrophic factor (BDNF) have been suggested as survival factors for midbrain dopaminergic neurons, a group of neurons primarily affected in Parkinson disease.

To investigate the physiological requirements for GDNF and BDNF to establish and maintain an important output pathway of these neurons—the nigrostriatal pathway—in the intact brain, we generated mutant mice with regionally selective ablations of the receptors for these survival factors, Ret (receptor of GDNF and related family members) or TrkB (BDNF receptor). Surprisingly, these mice survive to adulthood and show normal development and maturation of the nigrostriatal system. However, in ageing mice, ablation of Ret leads to a progressive and cell-type–specific loss of substantia nigra pars compacta neurons and their projections into the striatum. Our findings establish Ret and subsequent downstream effectors as critical regulators of long-term maintenance of the nigrostriatal system.

## Abstract 3

Ret, a receptor for glial cell line-derived neurotrophic factor, selectively regulates long-term maintenance of the nigrostriatal dopaminergic system.

## Introduction

The ventral mesencephalon contains the majority of dopaminergic (DA) neurons in the vertebrate brain with important functions for maintaining the mental and physical health of the organism. They form two prominent pathways: DA neurons of the substantia nigra pars compacta (SNpc) extend their axons mainly into the dorsal striatum (caudate-putamen) to form the nigrostriatal pathway essential for the control of voluntary motor behavior. DA neurons of the ventral tegmental area (VTA) project their fibers mostly into the ventral striatum (nucleus accumbens), olfactory tubercle, septum, amygdala, hippocampus, and cortex collectively referred to as the mesocorticolimbic system. This system has important function in controlling emotion-based behavior such as motivation and reward. Pathological changes in the DA systems result in psychosis, schizophrenia, attention deficit/hyperactivity disorder (ADHD), depression, addiction, and, most prominently, Parkinson disease (PD).

PD is the most common neurodegenerative movement disorder, clinically characterized by resting tremor, rigidity, postural imbalance, and bradykinesia. The underlying pathological event in PD is the progressive loss of DA neurons in the SNpc, often accompanied by intracytoplasmic proteinaceous inclusions termed Lewy bodies [1] and by neuroinflammatory processes [2]. Because of presymptomatic compensation [3], behavioral symptoms appear by a threshold effect, when 50%–60% of SNpc neurons and 70%–80% of striatal dopamine are lost [4,5]. Healthy individuals also experience continuous loss of DA neurons, but they remain asymptomatic as long as the critical threshold is not reached. The questions about the molecular etiology of PD and the selective neuronal vulnerability have not been answered satisfactorily.

Endogenous neurotrophic factors regulate natural cell death during development and maintain target innervations and cell survival during postnatal life. Declining production of a neurotrophic factor or impaired signal transduction in ageing neurons may contribute to pathological neurodegeneration [6]. Glial cell line–derived neurotrophic factor (GDNF) is a member of the GDNF family of neurotrophic factors that signal through a two-component receptor complex consisting of the Ret (rearranged during transfection) receptor tyrosine kinase and the GPI-linked GDNF family receptor alphas (GFRα) [7]. GDNF was suggested to be a target-derived neurotrophic factor for developing DA neurons [8] and a postnatal survival factor for midbrain DA neurons (reviewed in [9,10]). Genetic evidence, however, is limited, because *GDNF* and *Ret* null mutant mice die at birth [10]. GDNF protects DA neurons from the effects of neurotoxins such as 1-methyl-4-phenyl-1,2,3,6-tetrahydropyridine (MPTP) (reviewed in [7,11]). GDNF is currently tested in clinical trials (using different delivery systems) with the hope that it will ameliorate PD symptoms, but the results are so far conflicting [12,13]; see also [14] and [15].

Brain-derived neurotrophic factor (BDNF) is a member of the neurotrophin family and signals through the TrkB receptor tyrosine kinase and the p75 receptor. BDNF and TrkB are widely expressed throughout the adult and ageing brain, including midbrain DA neurons and the striatum [16,17], but age- and PD-related decreases in the expression of BDNF and reduced responsiveness to BDNF have been observed (reviewed in [6,18]). DA neuron loss after BDNF ablation during development [19] suggested that impaired signaling through TrkB may compromise DA neuron survival. BDNF modulates nigrostriatal functions and rescues DA neurons in PD animal models [9,20,21]. *BDNF* and *TrkB* null mutant mice do not survive to adulthood, preventing the genetic analysis of their roles in long-term DA neuron survival [22,23].

To investigate the physiological requirements for Ret and TrkB to establish and maintain the nigrostriatal pathway, we generated mice with regionally selective *Ret* and *TrkB* ablations that are compatible with postnatal survival of the mice. We find that Ret, but not TrkB, regulates long-term maintenance of the nigrostriatal DA system. *Ret* ablation causes progressive and late loss of DA neurons in SNpc, degeneration of DA nerve terminals in striatum, pronounced reactive gliosis, microglial activation, and reduced levels of evoked dopamine release. Together, these data establish Ret as an important signaling receptor for nigrostriatal DA system preservation and suggest conditional Ret mutants as an interesting model to study presymptomatic compensatory mechanisms in this system and early PD-related pathologies.

## Results

### Generation of Mice Lacking Ret and TrkB Receptors in SNpc DA Neurons

**Figure 1:**
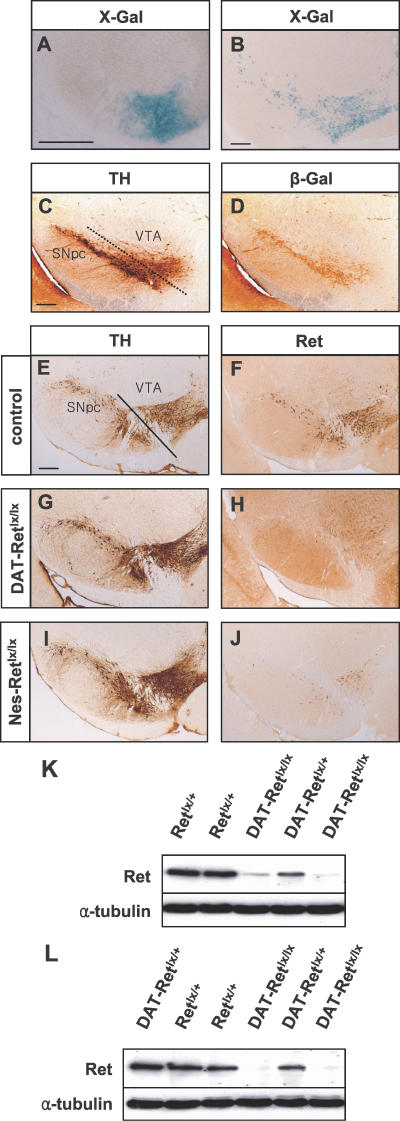
Conditional Ablation of Ret Expression in the Nigrostriatal System (A–D) Recombination efficiency of *DAT-Cre* mice crossed with *Rosa26R* lacZ reporter mice (DAT-Rosa26R). β-galactosidase (X-Gal) activity (blue) in coronal brain sections of DAT-Rosa26R transgenic mice at embryonic day E15.5 (A) and at 3-mo postnatal (B). Anti- TH (C) and anti–β-galactosidase (β-Gal) (D) immunostaining in adjacent brain sections of 2-y-old DAT-Rosa26R mice. Cre activity is restricted to SNpc and VTA. (E–J) Immunohistochemical detection of TH (E, G, and I) and Ret (F, H, and J) in adjacent coronal brain sections of 3-mo-old wild-type (wt), *DAT-Ret^lx/lx^,* and *Nes-Ret^lx/lx^* mice. Note the nearly complete removal of Ret immunoreactivity in SNpc and VTA of *DAT-Ret^lx/lx^* and *Nes-Ret^lx/lx^* mice. (K and L) Western blot analysis of Ret protein levels in protein lysates from SNpc (K) and striatum (L) of 3-mo-old control *(Ret^lx/+^*and *DAT-Ret^lx/+^)* and *DAT-Ret^lx/lx^* mutant mice. Immunoblots were reprobed with anti–α-tubulin antibodies as loading controls.

To disrupt the genes encoding Ret and TrkB in a regionally selective manner, we used mice with floxed alleles of *Ret* [24] and *TrkB* [25] in combination with *Dopamine transporter (DAT)-Cre* mice [26] (*DAT-Ret^lx/lx^* and *DAT-TrkB^lx/lx^* mice, respectively) and *Nestin-Cre* mice [27] (*Nes-Ret^lx/lx^* mutants). *DAT-Cre* mice had been generated by knocking Cre into the 5′ UTR region of the endogenous mouse *DAT* locus [26], whereas *Nestin-Cre* mice express Cre from a transgene [27]. For *DAT-Cre* mice, it was previously shown that virtually all (95%) of tyrosine hydroxylase (TH)-positive cells in SNpc and the nearby VTA regions express Cre and show Cre-mediated recombination in adult mice, whereas weak lacZ reporter activity was seen in DA neurons of the olfactory bulb and hypothalamus. No reporter activity was seen in the striatum [26]. We confirmed these observations and extended the analysis to other time points, including embryonic day 15 and 2-y postnatal (Figure 1A–1D). Our results indicate that DAT-Cre–mediated recombination is region selective from late embryonic stages to aged mice. Ret expression is high in the SNpc and VTA of adult control mice (Figure 1E and 1F) and is efficiently removed in *DAT-Ret^lx/lx^* and *Nestin-Ret^lx/lx^* mice (Figure 1G–1J). Western blot analysis of Ret protein revealed a nearly complete loss of the protein in SN of *Dat-Ret^lx/lx^* mice, and complete loss of Ret in the striatum of *DAT-Ret^lx/lx^* mice (Figure 1K and 1L). TrkB expression is found in nigral DA neurons [17], but also in other neuronal populations in the entire ventral midbrain (unpublished data). The conditional *trkB^lx^* allele has previously been used for region-specific removal of TrkB in several studies [25,28], indicating that this locus can efficiently be modified by Cre recombination. We were unable to visualize loss of TrkB by immunostaining and Western blotting in *DAT-TrkB^lx/lx^* mice, because the TH-positive subpopulation expresses low amounts of TrkB and is a minor population within the TrkB expression domain. However, we detected the recombined *TrkB^lx^* allele by PCR specifically in SNpc, but not in striatum, of *DAT-TrkB^lx/lx^* mice (Figure S1A). In addition, using laser capture microdissection combined with single-cell RT-PCR, we found a 65% decrease of TrkB mRNA–positive DA neurons in the SNpc in *DAT-TrkB^lx/lx^* mice compared to controls (Figure S1B–S1D).

### Substantial Loss of Midbrain DA Neurons in Aged *DAT-Ret^lx/lx^* Mice

**Figure 2:**
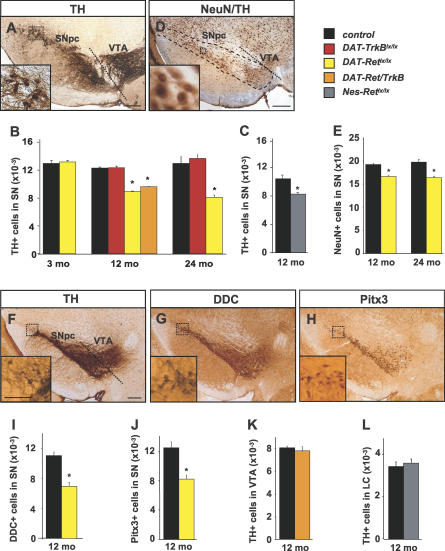
Progressive Loss of Nigral DA Neurons in *DAT-Ret^lx/lx^* Mice (A) Coronal brain section of a 3-mo-old wild-type mouse showing DA neurons in the SNpc and the VTA stained with an antibody against TH. The inset shows a higher magnification view of the stippled area. (B and C) Stereological quantification of TH-positive DA neurons in the SNpc of 3-, 12-, and 24-mo-old control, *DAT-TrkB^lx/lx^, DAT-Ret^lx/lx^,* and double homozygous *Dat-Ret/TrkB* mice (C) (*n* = 3 mice per genotype), *Nes-Ret^lx/lx^* mutant mice and littermate controls (D) (*n* = 4 mice per genotype). *, *p* < 0.05 (Student *t*-test). (D) Double immunostaining for NeuN and TH (very mild staining protocol to outline the SNpc [stippled area]). The inset shows a higher magnification view of the stippled box, displaying nuclear localization for NeuN and cytoplasmic immunoreactivity for TH. (E) Stereological quantification of NeuN-positive neurons in the SNpc of 12- and 24-mo-old control and *DAT-Ret^lx/lx^* mice (*n* = 5 mice per genotype at 12 mo, and *n* = 4 mice per genotype at 24 mo). *, *p* < 0.0001 and *p* < 0.001 for 12- and 24-mo-old *DAT-Ret^lx/lx^* mice, respectively (Student *t*-test). (F–H) Adjacent sections of SNpc and VTA of a 1-y-old wild-type mouse stained for TH (F), dopa-decarboxylase (G), and Pitx3 (H). Insets show higher magnification images. (I and J) Stereological quantification of DDC-positive (I) and Pitx3-positive (J) cells in the SNpc of 12-mo-old littermate control and *DAT-Ret^lx/lx^* mice (*n* = 3 mice per genotype). *, *p* < 0.05 (Student *t*-test). (K and L) Stereological quantification of TH-positive cells in the VTA region of 1-y-old control and *DAT-Ret/TrkB* mutant mice (K) (*n* = 3 mice per genotype; *p* > 0.5; Student *t*-test) and in the LC of 12-mo-old control and *Nes-Ret^lx/lx^* mutant mice (L) (*n* = 4 mice per genotype; *p* >0.5; Student *t*-test). Scale bar indicates 250 μm and, in insets, 100 μm.

*DAT-Ret^lx/lx^* and *DAT-TrkB^lx/lx^* knock-outs are viable and fertile. To detect morphological alterations in the nigrostriatal system, brain tissue sections of mutant and control mice (floxed Ret and/or TrkB mice; heterozygote DAT-Cre mice) were immunostained for TH and subjected to stereological quantification (Figure 2A). A significant decrease of approximately 25% in the number of TH-positive neurons in the SNpc was found in 1-y-old *DAT-Ret^lx/lx^,* but not *DAT-TrkB^lx/lx^* mice, compared to age-matched control mice (Figure 2B). Similar reductions of SNpc neurons were also observed in a more widespread knock-out of *Ret* using *Nestin-Cre* mice (Figure 2C). Combined loss of *Ret* and *TrkB* did not significantly enhance the loss of TH-positive neurons, suggesting that TrkB signaling has a minor, if any, role in the survival of a subset of nigral DA neurons (Figure 2B). A time-course analysis starting at 3 mo of age revealed that the nigrostriatal system of *DAT-Ret^lx/lx^* mutants had developed normally, despite the fact that DAT-Cre–mediated recombination started during late embryogenesis (Figure 2B). At 2 y of age, *DAT-Ret^lx/lx^* mice have lost 38% of TH-positive neurons, compared to age matched control mice (Figure 2B), whereas in 2-y-old *DAT-TrkB^lx/lx^* mice, no reduction of TH-positive neurons was detected (Figure 2B). We used the general neuronal marker NeuN and additional independent markers of nigral DA neurons to further characterize the defects in *DAT-Ret^lx/lx^* mutants. Anti-NeuN immunostaining combined with a weak TH staining to visualize the SNpc revealed 14% and 17% loss of NeuN-positive cells in the SNpc of 1-y-old and 2-y-old *DAT-Ret^lx/lx^* mutants, respectively (Figure 2D and 2E). Nissl staining, which labeled approximately five times more cells than TH staining, did not reveal significant changes in *DAT-Ret^lx/lx^* mutants (Figure S2A–S2C). Anti–dopa-decarboxylase (DDC) and anti-Pitx3 immunostaining [29] revealed reductions of 35%–40% of immunopositive nigral DA neurons in *DAT-Ret^lx/lx^* mutants compared to controls (Figure 2F–2J). In summary, the data suggest that loss of Ret leads to the loss of neurons rather than to reduced TH expression in a subpopulation of the SNpc. The observed defects in *Ret* mutants were region specific: The nearby VTA region was not affected in *DAT-Ret^lx/lx^* mice, and the locus coeruleus (LC) was not affected in *Nes-Ret^lx/lx^* mutants (despite the fact that Nestin-Cre recombines in and Ret is expressed in the LC) (Figure 2K and 2L). The loss of SNpc DA neurons in PD is often associated with the formation of α-synuclein–containing aggregates, so-called Lewy bodies. We were not able to detect accumulation or aggregates of α-synuclein in the cell bodies of *Ret* or *TrkB* or double-mutant mice (Figure S3A–S3I and unpublished data).

### Loss of DA Nerve Terminals in the Striatum of Aged *DAT-Ret^lx/lx^* Mutants

**Figure 3:**
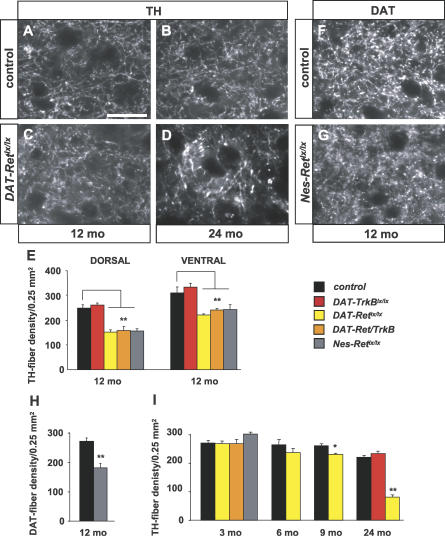
Progressive Loss of Striatal Innervation in *DAT-Ret^lx/lx^,* but Not *DAT-TrkB^lx/lx^* Mice (A–D, F, and G) Representative images of dorsal striatum stained by immunofluorescence using antibodies against TH (A–D) and DAT (F and G) of control (A, B, and F) and *DAT-Ret^lx/lx^* mutants (C, D, and G) at 12 (A, C, F, and G) and 24 mo of age (B and D). (E) The innervation density based on anti-TH immunofluorescence was quantified in dorsal versus ventral striatum of 12-mo-old controls (*n* = 16) versus *DAT-TrkB^lx/lx^* (*n* = 4), *DAT-Ret^lx/lx^* (*n* = 6), double *DAT-Ret/TrkB* (*n* = 5), and *Nes-Ret^lx/lx^* mutants (*n* = 7). *DAT-Ret^lx/lx^,* double *DAT-Ret/TrkB,* and *Nes-Ret^lx/lx^* mutants showed significant reductions in TH fiber density in dorsal (*p* < 0.001) and ventral striatum (*p* < 0.001, *p* < 0.01, and *p* < 0.01 for *DAT-Ret^lx/lx^,* double *DAT-Ret/TrkB,* and *Nes-Ret^lx/lx^* mutants, respectively). **, *p* < 0.01 (Student *t*-test). (H) The innervation density based on anti-DAT immunofluorescence was quantified in 12-mo-old *Nes-Ret^lx/lx^* mutants compared to age-matched controls (*n* = 4 per genotype; *p* < 0.001, Student *t*-test). (I) Time course of TH-positive fiber loss from 3 to 24 mo of age. *DAT-Ret^lx/lx^* mutant mice show a progressive fiber loss, starting at 6 to 9 mo (*p* = 0.09 and *p* < 0.05 at 6 mo and 9 mo, respectively) and maximizing at 24 mo (*p* < 0.0001). *DAT-TrkB^lx/lx^* mutant mice do not show any signs of fiber loss even at 24 mo of age (*p* = 0.13). *, *p* < 0.05; **, *p* < 0.01 (Student *t*-test). Scale bar indicates 25 μm.

We next sought to evaluate the possibility that Ret and TrkB signaling would be required for maintenance of target innervation of nigral DA neurons. The quantification of TH-positive fiber density revealed an approximately 40% decrease in the dorsal striatum of 1-y-old *DAT-Ret^lx/lx^* mice, compared to age-matched controls (floxed Ret and/or TrkB mice; heterozygote DAT-Cre mice) or *DAT-TrkB^lx/lx^* knock-outs (Figure 3A–3E). Similar reductions in TH fiber density (38%) were seen in 1-y-old *DAT-Ret/TrkB* double knock-outs and in *Nestin-Ret^lx/lx^* mutants (Figure 3E). The reduction was somewhat more pronounced in dorsal versus ventral striatum (40% versus 28% in *DAT-Ret^lx/lx^* mice, and 38% versus 20% in *DAT-Ret/TrkB* double-mutant mice and *Nestin-Ret^lx/lx^* mutants), correlating with the innervation preference by nigral DA neurons [30] (Figure 3E, ventral). To exclude the possibility that the reduction of TH fiber density reflected a reduction of TH protein, rather than fibers, we used DAT protein as an independent and selective marker for DA terminals. Because the aged DAT-Cre knock-in mice have reduced levels of DAT protein (unpublished data) due to the loss of one functional copy of the *DAT* gene, we analyzed *Nes-Ret^lx/lx^* instead and found a similar reduction of DAT fiber density in mutants versus control mice (Figure 3F–3H). A time-course analysis between 3 mo and 2 y revealed that this was an age-dependent process that started at around 9 mo of age and was most pronounced in 2-y-old mice (63% reduction in *DAT-Ret^lx/lx^* mice versus controls) (Figure 3I). In conclusion, Ret signaling is required for maintenance of target innervation of midbrain DA neurons.

### Non-Cell Autonomous Dysfunction of Striatal Neurons in Aged *DAT-Ret^lx/lx^* Mutants

**Figure 4:**
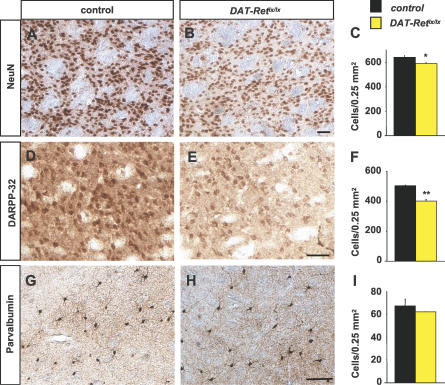
Loss of Postsynaptic Target Cells but Not Local Striatal Interneurons in *DAT-Ret^lx/lx^* Mice Immunohistochemical stainings of dorsal striatum of 2-y-old control (A, D, and G) and *DAT-Ret^lx/lx^* mutants (B, E, and H) for NeuN (A and B), DARPP-32 (D and E), or parvalbumin (G and H). Histograms showing the number of NeuN-positive (C), DARPP-32–positive (F), and parvalbumin-positive cells (I) in *DAT-Ret^lx/lx^* mutants and age-matched controls (*n* = 3–5 each genotype). Note also the reduced staining intensities for NeuN and DARPP-32 in *DAT-Ret^lx/lx^* compared to control mice. *, *p* < 0.05; **, *p* < 0.01 (Student *t*-test). Scale bars indicate 50 μm.

We next asked if loss of nigrostriatal innervation by midbrain DA neurons would cause non-cell autonomous dysfunction of striatal target neurons that do not express Ret. Staining with the neuronal marker NeuN, which detects all striatal neurons including interneurons, revealed a decreased staining intensity and a small, but significant reduction of NeuN-positive cells (8%; *n* = 5 for each group; *p* < 0.05, Student *t*-test) in 2-y-old *DAT-Ret^lx/lx^* compared to age-matched control striatum (Figure 4A–4C). More importantly, expression of DARPP-32 (dopamine and cyclic adenosine 3′,5′-monophosphate-regulated phosphoprotein, 32 kDa), a protein expressed by nearly all dopaminoceptive striatal projection neurons [30], was also reduced and the number of positive cells decreased by 20% (*n* = 3 for each group; *p* < 0.01, Student *t*-test) in *DAT-Ret^lx/lx^* mutants compared to controls, suggesting the existence of postsynaptic dysfunction including unhealthy, atrophic cells and perhaps cell loss as consequence of Ret ablation in DA neurons (Figure 4D–4F). In contrast, expression of the calcium-binding protein parvalbumin, which labels local GABAergic interneurons, was not reduced in *DAT-Ret^lx/lx^* mutants compared to controls (Figure 4G–4I). These results suggest that loss of nigrostriatal innervation indirectly affects a fraction of dopaminoceptive striatal neurons.

### Gliosis in Dorsal Striatum of *DAT-Ret^lx/lx^* Mice

**Figure 5:**
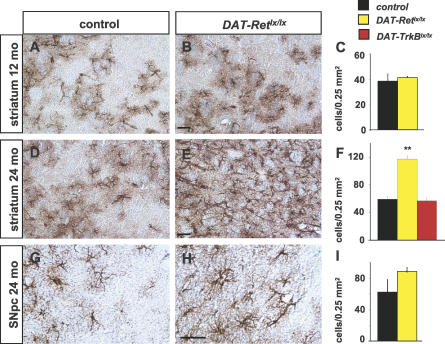
Gliosis in Dorsal Striatum of *DAT-Ret^lx/lx^* Mice (A, B, D, E, G, and H) Bright-field photomicrographs of dorsal striatum (A, B, D, and E) and SNpc (G and H) of 12-mo-old (A and B) and 24-mo-old (D, E, G, and H) control (A, D, and G) and *DAT-Ret^lx/lx^* mutants (B, E, and H) stained for GFAP. (C, F, and I) Histograms showing the number of GFAP-positive reactive astrocytes (*n* = 3–5 per genotype). There is a 2-fold increase in the number of reactive astrocytes in the striatum of 2-y-old *DAT-Ret^lx/lx^* mutants as compared to wild-type controls and *DAT-TrkB^lx/lx^* mutants (F) (*p* < 0.0001), whereas no difference is seen in 12-mo-old *DAT-Ret^lx/lx^* mutants compared to controls (C) (*p* = 0.9). No significant increase in the number of reactive astrocytes is seen in the SNpc of 24-mo-old *DAT-Ret^lx/lx^* mutants compared to controls (I) (*p* = 0.24). **, *p* < 0.01 (Student *t*-test). Scale bars indicate 50 μm.

Having established substantial loss of nigrostriatal innervation and some striatal dysfunction in *DAT-Ret^lx/lx^* mutants, we next asked if these degenerative processes would cause gliosis by invading reactive astrocytes. We used immunoreactivity against glial fibrillary acidic protein (GFAP) as an indicator of the astroglial response to genetically induced DA nerve terminal damage (Figure 5). Staining of 2-y-old brains revealed a massive reactive gliosis in dorsal striatum of *DAT-Ret^lx/lx^* mutants compared to controls (Figure 5D–5F; *n* = 5 mice per group, *p* < 0.0001, Student *t*-test). No increased GFAP immunoreactivity was observed in 2-y-old *DAT-TrkB^lx/lx^* striatum (Figure 5F; *n* = 4 per group, *p* < 0.0001, Student *t*-test), or in younger (12-mo-old) *DAT-Ret^lx/lx^* mutants (Figure 5A–5C; *n* = 3 mice per group, *p* = 0.90, Student *t*-test), or in other brain regions such as the neocortex of 2-y-old *DAT-Ret^lx/lx^* mutants (unpublished data). GFAP immunoreactivity in SNpc of 2-y-old *DAT-Ret^lx/lx^* mutants was not significantly enhanced compared to controls (Figure 5G–5I; *n* = 3 per group, *p* = 0.24, Student *t*-test) despite the marked loss of TH-positive cells in this structure. Because Ret is not genetically ablated in astrocytes, these results suggest that the gliosis in the striatum of *DAT-Ret^lx/lx^* mice is non-cell autonomously caused by degenerating DA nerve terminals.

### Inflammation in SNpc of *DAT-Ret^lx/lx^* Mice

**Figure 6:**
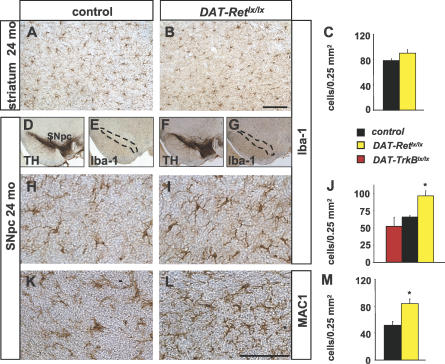
Inflammation in SNpc of *DAT-Ret^lx/lx^* Mice (A, B, D–I, K, and L) Immunohistochemical stainings of dorsal striatum (A and B) and SNpc (D–I, K, and L) of 24-mo-old control (A, D, E, H, and K) and *DAT-Ret^lx/lx^* mice (B, F, G, I, and L) for Iba-1 (A, B, E, G, H, and I), TH (D and F), and MAC1 (K and L). To localize microglial cells in SNpc, adjacent sections were stained for TH, and the area of the SNpc was marked and copied to the adjacent section stained for macrophages. (C, J, and M) Histograms showing the number of Iba-1–positive (C and J) and MAC1-positive (M) cells in the striatum (C) and SNpc (J and M) of 24-mo-old (C and J) *DAT-Ret^lx/lx^* mice and controls. No significant alterations in the numbers of Iba-1–positive cells were observed in the striatum of 24-mo-old mutants and controls ([C] *n* = 4, *p* = 0.065). A significant increase in the numbers of Iba-1–positive cells was observed in the SNpc of 24-mo-old *DAT-Ret^lx/lx^* mice compared to controls (J) (*n* = 5, *p* < 0.05). The same result was obtained using MAC1 as a second independent microglial marker (M) (*n* = 3, *p* < 0.05). *, *p* < 0.05 (Student *t*-test). Scale bars indicate 100 μm.

Inflammatory processes are often associated with and activated by a variety of neuronal insults including PD and Alzheimer disease. We used immunohistochemistry for ionized binding calcium adapter molecule (Iba)-1 to detect microglia in brains of *DAT-Ret^lx/lx^* mice. The numbers of Iba-1 immunopositive cells were not significantly increased in dorsal striatum of 2-y-old *DAT-Ret^lx/lx^* mice compared to controls (Figure 6A–6C; *n* = 4 mice per group, *p* = 0.065, Student *t*-test). In contrast, we observed an approximately 45% increase in the number of Iba-1 immunopositive cells in SNpc of 2-y-old *DAT-Ret^lx/lx^* mice compared to controls and *DAT-TrkB^lx/lx^* mice (Figure 6D–6J; *n* = 3 mice for *DAT-TrkB^lx/lx^* and *n* = 5 mice for controls and *DAT-Ret^lx/lx^*, *p* < 0.05, Student *t*-test). Similar results were obtained using macrophage antigen alpha (MAC1, CD11b, or CR3) as a second, independent marker (Figure 6K–6M; *n* = 3 mice per group, *p* < 0.05, Student *t*-test). No differences in the numbers of Iba-1–positive microglial cells were detected in 1-y-old *DAT-Ret^lx/lx^* mice compared to controls (unpublished data). Similar to reactive astrocytes, the *Ret* gene was not subjected to recombination in microglia of *DAT-Ret^lx/lx^* mice, suggesting that the neuroinflammation occurred as a result of neuronal cell death.

### Reduced Dopamine Release in the Striatum of *DAT-Ret^lx/lx^* Mice

**Figure 7:**
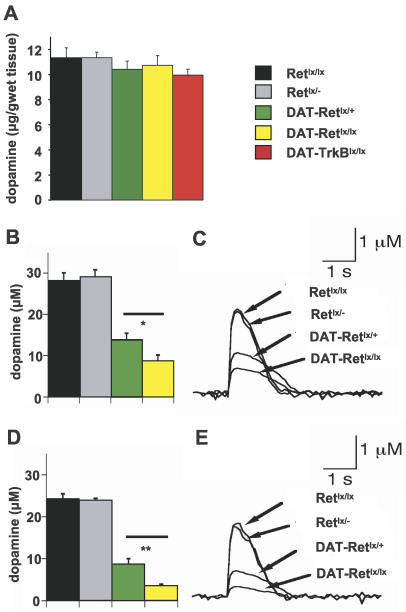
Reduced Dopamine Release in the Striatum of *DAT-Ret^lx/lx^* Mice (A) Total dopamine levels normalized to 2,3-dihydroxybenzoic acid (DHBA) and expressed relative to the weight of wet striatum (grams) of 2-y-old control mice *(Ret^lx/lx^),* heterozygous *Ret^lx/−^,* heterozygous *DAT-Ret^lx/+^,* homozygous *DAT-Ret^lx/lx^,* and *DAT-TrkB^lx/lx^* mice. Note the minor reduction of total dopamine levels in all mice carrying the DAT-Cre knock-in construct. (B–E) Evoked dopamine release after electrical stimulation in the dorsal striatum of control mice (*Ret^lx/lx^* and *Ret^lx/−^* mice), heterozygous *DAT-Ret^lx/+^* mice, and homozygous *DAT-Ret^lx/lx^* mice of 1 y (B and C) or 2 y (D and E) of age. In both age groups, there is a significant decrease of released dopamine in the mice carrying the DAT-Cre knock-in construct compared to controls. There is a further significant decrease in the homozygous *DAT-Ret^lx/lx^* mice due to the lack of Ret (*n* = 5 per genotype, *p* < 0.05, Student *t*-test). *, *p* < 0.05; **, *p* < 0.01 (Student *t*-test). (C and E) Representative traces of single evoked dopamine release in different control and mutant mice.

PD is clinically defined by a decrease in dopamine levels that result in motor impairments. To determine the effects of nerve terminal loss in *DAT-Ret^lx/lx^* mice on the DA output capacity of the system, we measured total levels and evoked dopamine release in the striatum of mutant mice. Striatal levels of dopamine and one of its major metabolites, dihydroxyphenylacetic acid (DOPAC), were similar in *DAT-Ret^lx/lx^* and control mice at 3, 12, and 24 mo (Figure 7A and unpublished data). The somewhat lower values in all mice (mutants and controls) carrying the *DAT-Cre* transgene compared to *DAT-Cre–*negative controls is due to the reduced levels of DAT protein, which regulates dopamine transport and metabolism [31]. Evoked dopamine release was measured by fast-scan cyclic voltammetry (FSCV) following electrical stimulation of coronal slice preparations of mutant and control mice [32]. Electrical stimulation resulted in a stimulus intensity–dependent overflow of DA in the striatum. DA overflow is the result of released DA minus the DA reuptake by DAT. We observed a marked reduction of evoked DA overflow in the striatum of all mice carrying the *DAT-Cre* transgene (Figure 7B–7E) as described before [31]. Interestingly, the evoked DA overflow was further reduced in *DAT-Ret^lx/lx^* mice compared to *DAT-Ret^lx/+^*control mice in both 1-y-old and 2-y-old mutants (*n* = 5 per genotype, *p* < 0.05 and *p* < 0.01 for 1-y-old and 2-y-old mutants, respectively, one-way analysis of variance and post-hoc Student *t*-test). Together with the unchanged input–output curves of the FSCV experiment (Figure S4), these data suggest that the reduced dopamine release and reuptake in the Ret mutants is likely due to the reduced number of DA fibers in the striatum. To determine to what extent these histological and physiological alterations change the behavior of the *DAT-Ret^lx/lx^* mice, we tested *DAT-Ret^lx/lx^* and control mice for behavioral alterations in open-field and rotarod tasks, and in voluntary and forced swimming tasks (Protocol S1). The behavior was essentially unaffected in the mutants (Figure S5).

## Discussion

In the present study, we show that signaling by Ret and TrkB receptors is not essential for establishment of the nigrostriatal system. TrkB signaling appears to play a minor, if any, role in maintaining long-term cell survival or target innervation of midbrain DA neurons in aged mice. In contrast, Ret ablation leads to a progressive and cell-type–specific loss of SNpc neurons and their afferents with adult onset, with subsequent alterations in physiology and appearance of neuroinflammatory responses. These findings establish Ret and subsequent downstream effectors as critical regulators of long-term maintenance of the nigrostriatal DA system. Because similar alterations are observed in the early phases of PD, *DAT-Ret^lx/lx^* mice might be useful for gaining insights into the molecular mechanisms involved in the development of PD.

### Ret and TrkB Are Dispensable for the Development of the Nigrostriatal System

The apparently normal development and maturation of the nigrostriatal system in *DAT-Ret^lx/lx^, DAT-TrkB^lx/lx^,* and *DAT-Ret/TrkB* mice was rather surprising in light of the known in vitro neurotrophic effects of the respective ligands GDNF and BDNF on DA neurons [9]. Consistent with our results, transgenic overexpression of GDNF or knock-down of GDNF in mice transiently altered the number of DA neurons in the early postnatal days; however, these alterations did not persist into adulthood (see [8] and references within). Ablation of the *BDNF* gene in the developing mid-hindbrain region using Wnt1-Cre–mediated recombination resulted in reductions in the number of TH-positive neurons in the SN of newborn mice [19]. In contrast to our study, BDNF ablation was earlier, not cell-type specific, and more widespread (mid-hindbrain region), and may have caused alterations in other non-DA neurons and progenitor cells that influenced the development of the nigrostriatal system.

### Ret Is Required for Long-Term Maintenance of the Nigrostriatal System

We found that Ret is specifically required for long-term target innervation and cell survival of a significant fraction of SNpc DA neurons. In aged Ret mutant mice, the extent of target innervation loss exceeded the degree of cell loss. This is consistent with observations from PD patients and MPTP-treated animals, which led to the current model of a “dying back” process to explain the DA neuron degeneration [4]. The requirement for Ret appears to be topographically specific: The nigrostriatal pathway from SNpc to dorsal striatum was more dependent on Ret than the mesolimbic pathway from the VTA to ventromedial striatum. At first glance, this seemed surprising because VTA neurons were shown to be more responsive than SN neurons to overexpressed GDNF, and the number of VTA neurons, but not SNpc neurons, persistently increased to adulthood in these mice [33]. GDNF is the likely Ret ligand in this system, because it promotes neurite outgrowth and sprouting of adult midbrain DA neurons more efficiently than do related family members [9,34], and its expression is maintained at detectable levels in the adult [35]. This suggests that GDNF/Ret signaling might be limiting, but not essential, for VTA neurons.

Differences in sensitivity toward stresses between VTA and SNpc neurons have been described previously. For example, SNpc neurons are more sensitive than VTA neurons to 6-OH-dopamine treatment and overexpression of human α-synuclein [36,37]. The presence of functional Kir6.2, a K-ATP channel, promotes cell death of SNpc, but not VTA neurons in two chronic mouse models of DA degeneration [38]. Aphakia mice, deficient for the transcription factor Pitx3 with important function for the establishment of the DA cell fate, preferentially lose SNpc, but not VTA neurons [32]. These data suggest different physiological features of SNpc and VTA neurons in vivo, including possible differences in their cell death pathways and survival factor requirements. It is possible that other neurotrophic factors such as members of the TGF-β superfamily of cytokines [39] or MANF [40] are required for the survival of VTA neurons. Future genetic experiments will hopefully help to answer this question. Also, in PD patients and neurotoxin-based animal models of PD, the nigrostriatal pathway is most affected [4]. The reason for this specificity is not well understood, but it suggests that the molecular death pathways activated in PD, by MPTP and by loss of Ret, share similarities.

We also observed reduced levels of evoked dopamine release, but not of total dopamine amounts in the striatum of *DAT-Ret* mutants. With respect to total dopamine tissue content, it appears that reductions are generally observed when the majority of SNpc neurons are lost. For example, the recently published *En1^+/−^;En2^−/−^* mutant mice [41] show more than 60% loss of SNpc neurons and a 39% reduction in total striatal dopamine content. The *DAT-Ret* mutants do not show such a strong decrease in SNpc neurons and, not surprisingly, have normal levels of total dopamine content. In contrast to the *En* mutants, *DAT-Ret* mutants lose neurons during ageing which leaves more time for compensatory mechanisms (*En* mutants lose SNpc neurons before P15). The physiologically more important reduction in dopamine release after electric stimulation parallels the age-dependent loss of striatal innervation. It also parallels changes in postsynaptic neurons such as the decrease of DARPP-32 expression. Whether or not postsynaptic neurons become atrophic or eventually die in PD patients and animal models is not clear and has not been studied in great detail. There is evidence that loss of DA neurons in the basal ganglia or dopamine depletion can lead to changes in the striatum, such as loss of spines and glutamatergic synapses [42] and may eventually lead to cell death [43]. The mild postsynaptic alterations observed in the *DAT-Ret^lx/lx^* mice are consistent with the idea that presynaptic decrease in DA fibers can indirectly lead to pathological changes in postsynaptic striatal neurons.

Apoptosis contributes to PD neuronal loss [44] and is the predominant cell death mechanism of neurotoxin-based models using prolonged administration of low doses of MPTP, but the detection of apoptotic cells is difficult because of the very low frequency of dying cells and their rapid clearance from the tissue [4]. Apoptosis is most probably the cell death mechanism underlying neurotrophic factor deprivation; although, due to the late and selective degeneration of nigral DA neurons, we were unable to detect apoptotic cells in *DAT-Ret^lx/lx^* mice. What may the signaling pathways downstream of Ret be that maintain target innervation and mediate cell survival? Besides the well-documented importance of the PI3K/AKT pathway for neuronal survival in response to GDNF [7], GDNF also increases dopamine release and influences synaptic transmission [45,46], and might thereby influence the vulnerability of SNpc neurons. Axon degeneration often begins with breakdown of microtubules whose assembly and disassembly is regulated by microtubule-associated proteins (MAPs) and may involve collapsin-response-mediator-protein-2 (CRMP2). The expression of CRMP2 is induced by GDNF [47], and CRMP2 promotes axon growth and branching as a partner for tubulin heterodimers [48]. Work is currently in progress to investigate the role of downstream mediators of Ret in maintaining DA afferents and cell survival.

### The Role of TrkB in the Nigrostriatal System

Using single-cell RT-PCR, we detected TrkB mRNA in only 50% of nigral DA neurons. Ablation of TrkB expression in the majority of the TrkB^+^;TH^+^ pool in the SNpc did not cause any morphological alterations. Moreover, under sensitized conditions in the absence of GDNF/Ret signaling, the additional reduction of TrkB did not cause significant alterations beyond those evoked by lack of Ret alone. From these results, we conclude that the physiological role of TrkB in the nigrostriatal system is minor at best. Cell and fiber loss reported in previous studies using nonconditional alleles of *TrkB* [49,50] have to be interpreted with care, since several TrkB-positive cell types in this region are not DA. The discrepancies between constitutive and conditional mutants may be partially due to non-cell autonomous effects from cells outside the SNpc. Additional Cre lines and markers for specific cell populations will have to be employed to settle the issue completely.

### Glial Responses in Ageing Ret-Deficient Mice

Activated glial cells (astrocytes and microglia) have been associated with central nervous system (CNS) injuries and PD [2,51]. It is a matter of debate, whether activated microglial cells are neuroprotective by the release of trophic factors or participate in the propagation of neurodegenerative processes. The mechanisms that lead to microglial and astroglial cell responses in PD patients are not understood. Here, we found that Ret ablation resulted in gliosis in the striatum and to microglial activation in the SNpc. What could be the underlying mechanisms? First, degenerating axons may be a stronger signal for astrocytes than for microglia. Such a conclusion is based on previous studies on injured CNS axons, including peripheral motor axons. It was found that astrocytes participated in the removal of presynaptic boutons, whereas microglial participation was not required for this process (reviewed in [52]). Second, apoptotic CNS neurons may be sending signals that preferentially activate microglia. Previous studies have shown that axotomy of retinal ganglion cells in adult rats leads to protracted degeneration that can be delayed by the application of compounds that suppress macrophage and microglia activity, suggesting that the microglial system has a key role in eliminating severed neurons in the CNS [53]. Third, there may be intrinsic differences between striatum and SNpc that result in gliosis versus microglia activation. In MPTP-treated mice, the astrocytic reaction is consecutive to death of neurons, and astrocyte accumulation is observed primarily in the striatum rather than in the SNpc [44,54]. Likewise, in PD patients, astroglial responses are generally weak and microglial responses are dramatic in the SNpc; they culminate in subregions that are most affected by the neurodegenerative process (reviewed in [55]).

### Are *DAT-Ret^lx/lx^* Mice a Useful Genetic Model for Nigrostriatal Pathologies?

The nigrostriatal pathologies following Ret ablation display several features of presymptomatic PD including (1) specific and progressive degeneration of the nigrostriatal pathway, with adult onset (so far unique among genetic PD models), (2) greater loss of DA neurons in SNpc than in VTA, (3) greater degeneration of DA nerve terminals in dorsal than ventral striatum, (4) the presence of substantial neuroinflammation and gliosis, and (5) reduced levels of evoked dopamine release in striatum.

However, our *DAT-Ret^lx/lx^* mice are not a perfect model of symptomatic PD since they lack several hallmarks of the disease, the first being the lack of cytoplasmic inclusions containing α-synuclein. This suggests that SNpc neuron cell death occurs in the absence of α-synuclein aggregates similar to MPTP-based models and PD cases caused by parkin mutations [56]. The absence of behavioral deficits in *DAT-Ret^lx/lx^* mice could be explained by incomplete destruction of the nigrostriatal pathway below the reported threshold level for symptom appearance in human PD patients and the presence of compensatory mechanisms maintaining DA homeostasis [3]. The unaltered total amounts of striatal dopamine in the aged *DAT-Ret^lx/lx^* mice support the idea that these mice are still in a phase in which dopamine-dependent or -independent mechanisms stabilize the system. Genetic experiments are in progress to investigate whether the chronic GDNF deprivation stress in *DAT-Ret^lx/lx^* mice would make nigral DA neurons more susceptible to other cellular stresses ultimately leading to a more complete destruction of the nigrostriatal pathway. Preliminary data suggest that mild transgenic overexpression of human mutant Ala30Pro α-synuclein using the TH promoter did not aggravate the defects seen in *DAT-Ret^lx/lx^* mice (L. Aron, E. R. Kramer, P. Kahle, C. Haass, and R. Klein, unpublished data).

*DAT-Ret^lx/lx^* mutants may be useful in studies of age-related neurodegeneration. Neurotrophins are thought to improve the body's resistance to neurodegeneration. Environmental factors such exercise, dietary energy restriction, and cognitive stimulation protect neurons against dysfunctions and death. This may happen, in part, by induction of a mild stress response that induces the production of BDNF and GDNF [6]. Mice that lack neurotrophin responses in specific neuronal subpopulations should be excellent models to test these hypotheses. *DAT-Ret^lx/lx^* mutants could also be used for the identification of biomarkers associated with the first phases of nigrostriatal pathway degeneration. So far there is no evidence that PD can be caused by mutations in the GDNF and Ret gene because analysis of polymorphisms in the *GDNF* and *Ret* gene have not shown any association with PD [57]. But perhaps *GDNF/Ret* signaling is reduced as a secondary consequence in PD and leads to the increased vulnerability of SNpc neurons. Further experiments are required to clarify this issue. However, the physiological requirement of Ret signaling for the maintenance of the nigrostriatal system is an important issue, considering the potential for stem cell therapy to replace DA neurons in PD patients, and argues for further investigations toward optimizing the ongoing clinical trials using activators of the Ret pathway as potential therapy for PD.

## Materials and Methods

### Transgenic animals.

The generation of floxed *Ret (Ret^lx^)* [24], *TrkB (TrkB^lx^)* [25], and DAT-Cre [26] alleles, and the *Nestin-Cre* transgenic mice [27] was described previously. Cre mice were crossed with ROSA26R reporter mice [58] to test for proper Cre expression. The mice used in this study were kept on a C57Bl6/J genetic background with contributions of 129/sv from the embryonic stem cell culture and the different Cre mouse lines. Because both Cre lines show significant recombination in germ cells, the floxed allele derived from the parent that carries the Cre recombinase is often constitutively recombined; therefore, the homozygous Ret mutants carry one *Ret* allele recombined in a regionally specific manner and one *Ret* allele recombined constitutively. Unless specifically mentioned, the control mice for all experiments carried floxed alleles of *Ret (Ret^lx/lx^, Ret^lx/+^), TrkB (TrkB^lx/lx^, TrkB^lx/+^),* or one copy of Cre (DAT-Cre or Nestin-Cre).

### Histology and immunohistochemistry.

For β-galactosidase stainings, mice were perfused with PBS and 30% sucrose; for immunohistochemistry, mice were perfused with PBS and 4% paraformaldehyde. Subsequently, brains were removed from the scull, postfixed overnight in the same fixative, and cyroprotected by incubating them in 15% and 30% sucrose solutions. Left and right brain halves were embedded separately in egg yolk with 10% sucrose and 5% glutaraldehyde, and kept frozen at −80 °C until analyzed. The 30 μm–thick coronal sections were cut on a cryostat, collected free floating, and then directly used for stainings or stored in a cryoprotection solution at −20 °C until utilized. For fluorescent immunohistochemical stainings, sections were premounted; for all other stainings, free-floating sections were used. Primary antibodies used were goat anti-Ret (1:25; RDI, Flanders, New Jersey, United States, or Neuromics, North Field, Minnesota, United States), monoclonal mouse anti-tyrosine hydroxylase (1:2,000; DiaSorin, Stillwater, Massachusetts, United States), rabbit anti-dopa decarboxylase (1:100; Chemicon/Millipore, Billerica, Massachusetts, United States), monoclonal mouse anti–β-galactosidase (1:50; Sigma, St. Louis, Missouri, United States), rat anti-dopamine transporter (1:500; Chemicon/Millipore), rabbit anti-Pitx3 (1:1,000, provided by M. P. Smidt [29]), monoclonal mouse anti-NeuN (1:200; Chemicon/Millipore), rabbit anti-GFAP (1:500; DakoCytomation, Glostrup, Denmark), rabbit anti–DARPP-32 (1:50; United States Biological, Swampscott, Massachusetts, United States), monoclonal mouse anti-parvalbumin (1:10,000; Swant, Bellinzona, Switzerland), rabbit anti–Iba-1 (1:1,000; Wako, Neuss, Germany), monoclonal rat anti-MAC1 (1:200; Serotec, Kidlington, United Kingdom). For diaminobenzidine detection of the primary antibody, different Vectastain ABC kits (Vector Laboratories, Burlingame, California, United States) were used according to the provider's instructions. For NeuN/TH double labeling, we first stained for NeuN as described above, followed by a weak TH staining with more-diluted primary (1:20,000) and secondary antibodies (1:2,000) and avidin-HRP/biotin complexes (1:2,000). DA fiber density in the striatum was assessed on every third section spanning the striatum (between Bregma +1.10 mm and −0.10 mm) [59]. The mounted sections were blocked for 1 h in 5% BSA, 0.3% Triton X-100 in TBS, and incubated with the first antibody diluted in 2% BSA, 0.1%Triton X-100 in TBS at 4 °C overnight. The sections were washed three times in TBS for 5 min, incubated in biotinylated secondary antibody (1:200 anti-mouse or anti-rat, Vectastain) for 2 h at room temperature, again washed as described above, and treated with streptavidin-Cy3 (1:500; Sigma) for 2 h. After another three washing steps, sections were mounted in aqueous mounting medium with anti-fading reagent (Biomedia, Foster City, California, United States, or DakoCytomation), and pictures were taken with a fluorescent microscope (Axioplan; Zeiss, Göttingen, Germany) at 63×. For every section, three pictures in the dorsal striatum and two pictures in the ventral striatum were acquired. In order to automatically delineate the fibers and to increase the signal-to-noise ratio, the images were first thresholded and subsequently quantified with an automatic counting-grid macro implemented in the Metamorph software (Molecular Devices, Sunnyvale, California, United States). Stereological countings were done with the StereoInvestigator program (MicroBrightField, Williston, Vermont, United States) on every third section for the LC and at least every sixth section for the SNpc and VTA.

### Biochemistry.

For Western blot analysis from the substantia nigra and the striatum and for total dopamine measurements in the striatum, mouse brain tissue isolation was done by cutting 2 mm–thick coronal sections (2-mm rostral or caudal to the interaural line) from a freshly frozen brain and punching out 2-mm^2^ (substantia nigra) or 3-mm^2^ (striatum) tissue circles with sample corers (Fine Science Tools, Heidelberg, Germany). Western blot analysis was done according to standard techniques with a rabbit anti-Ret (1:250; Santa Cruz Biotechnology, Santa Cruz, California, United States) and a mouse monoclonal anti–α-tubulin antibody (1:500; Sigma). Total striatal dopamine was measured as described previously [60] with a few modifications.

### FSCV on brain slices.

Evoked release of dopamine was measured in 200 μm–thick coronal slices containing the striatum of control mice (*Ret^lx/lx^* and *Ret^lx/−^* mice), heterozygous *DAT-Ret^lx/+^* mice, and homozygous *DAT-Ret^lx/lx^* mice of 1 y or 2 y of age. The slice preparation was done as previously described [32]. Dopamine release was evoked by a single pulse (0–1,000 μA, 300 μs) applied through a bipolar stimulation electrode (bipolar stainless steel, 100 μm, insulated except for the tip) every 30 s. Dopamine was detected with 5-μm carbon-fiber disk electrodes insulated with electrodeposition paint (ALA Scientific Instruments, Westbury, New York, United States) using FSCV. Cyclic voltammograms (ramps from −500 mV to +1,000 mV and back to −500 mV versus a Ag/AgCl, 300 V/s) were repeated every 100 ms using an EPC10 amplifier (HEKA Electronic, Lambrecht, Germany). Stimulus-evoked dopamine overflow was measured by subtracting the background current obtained before stimulation (average of ten pre-stimulus responses) from the current measured after stimulation, using IgorPro software (Wavemetrics, Lake Oswego, Oregon, United States). The resulting voltammogram showed a typical dopamine profile, with an oxidation peak between 500 and 700 mV and a smaller reduction peak around −300 mV. The concentration of the dopamine overflow was calculated after calibrating the recording electrode in known concentrations of dopamine. The amount of dopamine released depends on the stimulation intensity, and the input–output relation was fitted with a sigmoid function *[dopamine]/[dopamine]_max_ = 1/{1 + exp[-(S.I. − S.I._1/2_)/k]}* where S.I. = stimulation intensity.

## Supporting Information

Figure S1 Recombination of the Floxed TrkB Locus in *DAT-TrkB* Mutant Mice (A) PCR results on genomic DNA from tissue of the SNpc and striatum (ST) of heterozygote *Nes-TrkB* mutant mice *(Nes-TrkB^lx/+^),* TrkB wild-type mice *(TrkB^+/+^),* and heterozygote *DAT-TrkB* mutant mice *(DAT-TrkB^lx/+^)* to detect the wild-type TrkB allele (TrkB wt), non-recombined floxed TrkB allele (TrkB lox), and the recombined floxed TrkB allele (TrkB lox rec). In *Nes-TrkB^lx/+^* mice, we detected a strong TrkB wt, a weak TrkB lox, and a strong TrkB lox rec band both in the SNpc and ST, because Nes-Cre efficiently recombines the floxed TrkB locus in most neurons. In *TrkB^+/+^* mice, we detected only the TrkB wt band. In *DAT-TrkB^lx/+^* mice, the PCR strongly amplified the TrkB wt and TrkB lox fragment from SNpc and ST genomic DNA. The TrkB lox rec band is only visible in the PCR reaction with the genomic DNA from SNpc, but not from ST, showing specific and efficient recombination of the floxed TrkB allele in DA neurons expressing Cre from the DAT locus.
(B) Coronal brain sections of the SNpc are shown before and after laser microdissection. In the area of the SNpc, large cells were selected, and single cells were collected for mRNA detection by RT-PCR.
(C) Representative results of the single-cell RT-PCR for TH and TrkB are shown from cells of control *(TrkB^+/+^)* and homozygous TrkB *(DAT-TrkB^lx/lx^)* mutant mice.
(D) Quantification of the RT-PCR results. In control mice, TrkB mRNA was detected in approximately 50% of TH-positive cells. This number was set to 100%. *DAT-TrkB^lx/lx^* mice showed a reduction of 65% of *TrkB;TH* double-positive cells.
(1.4 MB PDF)

Figure S2 Quantification of Nissl-Stained Cells in the SNpc of *DAT-Ret^lx/lx^* Mice (A) Coronal brain section of a 1-y-old wild-type mouse showing DA neurons in the SNpc and the VTA labeled for both Nissl (blue) and TH (brown).
(B) Higher magnification view of the boxed area of (A) showing the presence of numerous cells labeled only by Nissl and not by TH. TH staining was used to select the area for quantification.
(C) Stereological quantification of Nissl-stained cells in the SNpc of 1-y-old controls and *DAT-Ret^lx/lx^* mice (*n* = 4 mice per genotype, *p* = 0.17). No significant differences between the number of Nissl-positive cells in controls versus *DAT-Ret^lx/lx^* mice were seen.
(1.5 MB PDF)

Figure S3 No α-Synuclein Accumulation in SNpc of 2-Y-Old *DAT-Ret^lx/lx^*and *DAT-TrkB^lx/lx^* Mice Immunohistochemical detection of α-synuclein in SNpc using a purified sheep polyclonal antibody (α-syn (1)) or a crude rabbit antiserum (α-syn (2)). Both antibodies detected α-synuclein in SNpc of 3-mo-old transgenic mice (B and D) overexpressing α-synuclein under a TH promoter (TH-α-syn) but not in non-transgenic control littermates (control) (A and C). However, the crude rabbit antiserum previously used showed some unspecific cellular labeling (C) [49].
No α-synuclein accumulation was observed in 2-y-old control (E and H), *DAT-Ret^lx/lx^* (F and I), and *DAT-TrkB^lx/lx^* (G) mice. Again, the crude rabbit antiserum showed some unspecific staining (H and I). Scale bars indicate 100 μm.
(5.3 MB PDF)

Figure S4 Input–Output Curve of the FSCV Experiment The amount of dopamine release in the FSCV experiment of 1-y-old mice (A) and 2-y-old mice (B) depends on the stimulation intensity (SI). The input–output relation was fitted with a sigmoid function [DA]/[DA]max = 1/{1 + exp (SI − SI1/2)/k}. The stimulation intensity needed for half-maximal release in the striatum did not differ (12 mo: *Ret^lx/lx^* 48 ± 6 μA, *Ret^lx/−^* 52 ± 3 μA, *DAT-Ret^lx/+^* 50 ± 7 μA, and *DAT-Ret^lx/lx^* 54 ± 8 μA; 24 mo: *Ret^lx/lx^* 51 ± 5 μA, *Ret^lx/−^* 47 ± 10 μA, *DAT-Ret^lx/+^* 53 ± 4 μA, and *DAT-Ret^lx/lx^* 56 ± 9 μA), which suggests that the reduced DA output capacity in the Ret mutant mice is most likely due to the reduced fiber density and not to a presynaptic excitability function of Ret for regulating dopamine release.
Filled circle (•), *Ret^lx/lx^;* open circle (○), *Ret^lx/−^;* filled triangle (▴), *DAT-Ret^lx/+^;* open triangle (Δ), *DAT-Ret^lx/lx^*.
(298 KB PDF)

Figure S5 Behavioral Analysis of DAT-Ret Mice Mice older then 18 mo were tested in behavioral tests for general activity (A and B) and motor deficiencies (C and D). The vertical activity (rearing) of the mice was tested in open-field experiments (A); the horizontal activity was analyzed in a forced swimming test (B). The motor coordination was measured in a swimming tank (C) and a rotarod test (D) (*n* > 15).
(166 KB PDF)

Protocol S1 Supplementary Materials and Methods (56 KB DOC)

## Abbreviations

BDNF: brain-derived neurotrophic factor
CNS: central nervous system
DA: dopaminergic
FSCV: fast-scan cyclic voltammetry
GDNF: glial cell line–derived neurotrophic factor
GFAP: glial fibrillary acidic protein
Iba: ionized binding calcium adapter molecule
LC: locus coeruleus
PD: Parkinson disease
SNpc: substantia nigra pars compacta
TH: tyrosine hydroxylase
VTA: ventral tegmental area
