# Supplementary material for: Absence of Ret Signaling in Mice Causes Progressive and Late Degeneration of the Nigrostriatal System
Source: PLoS Biol. 2007 Feb 13;5(3):e39. doi: 10.1371/journal.pbio.0050039 (PMC1808500; doi:10.1371/journal.pbio.0050039)
Supplement: Supplementary file 1 [file pbio.0050039.sg001.pdf]

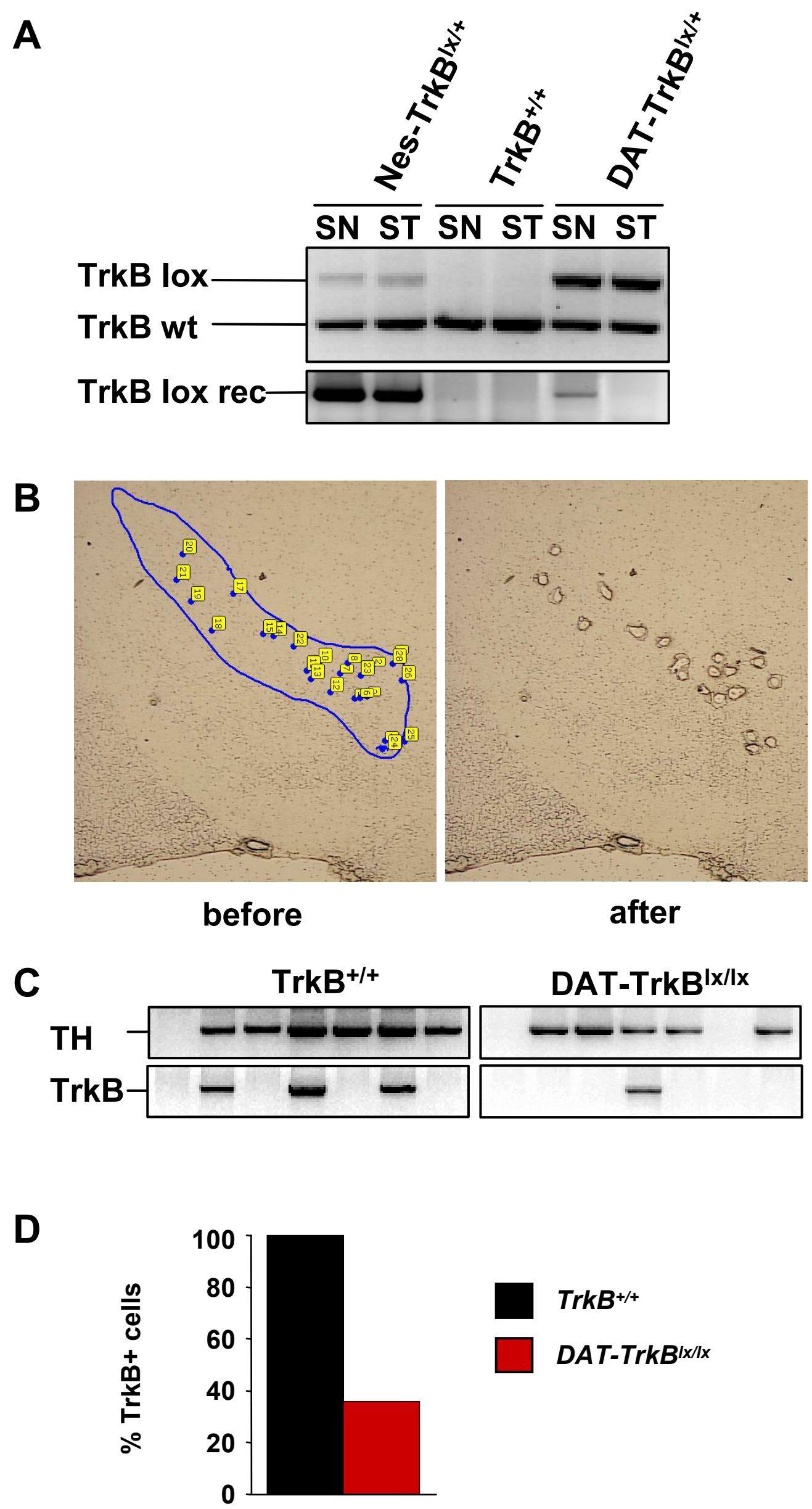
A
Nes-TrkB lx/+
TrkB +/+
DAT-TrkB lx/+
SN ST SN ST SN ST
TrkB lox
TrkB wt
TrkB lox rec
B
before
after
C
TrkB +/+
DAT-TrkB lx/lx
TH
TrkB
D
% TrkB+ cells
100
80
60
40
20
0
TrkB +/+
DAT-TrkB lx/lx
