# Supplementary material for: Absence of Ret Signaling in Mice Causes Progressive and Late Degeneration of the Nigrostriatal System
Source: PLoS Biol. 2007 Feb 13;5(3):e39. doi: 10.1371/journal.pbio.0050039 (PMC1808500; doi:10.1371/journal.pbio.0050039)
Supplement: Supplementary file 2 [file pbio.0050039.sg002.pdf]

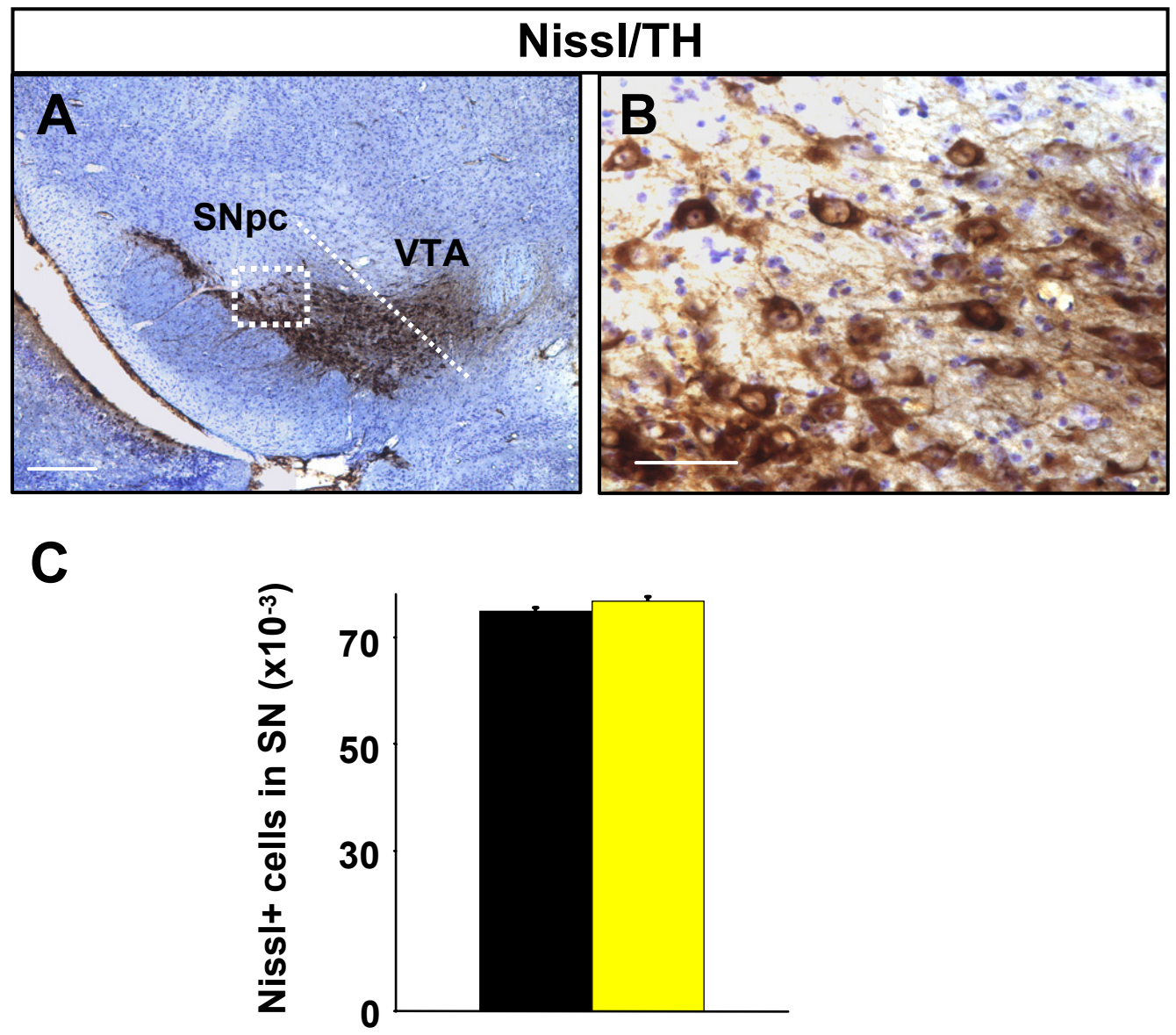
Nissl/TH
A
SNpc
VTA
B
C
Nissl+ cells in SN (x10-3)
70
50
30
0
