# Supplementary material for: Absence of Ret Signaling in Mice Causes Progressive and Late Degeneration of the Nigrostriatal System
Source: PLoS Biol. 2007 Feb 13;5(3):e39. doi: 10.1371/journal.pbio.0050039 (PMC1808500; doi:10.1371/journal.pbio.0050039)
Supplement: Supplementary file 3 [file pbio.0050039.sg003.pdf]

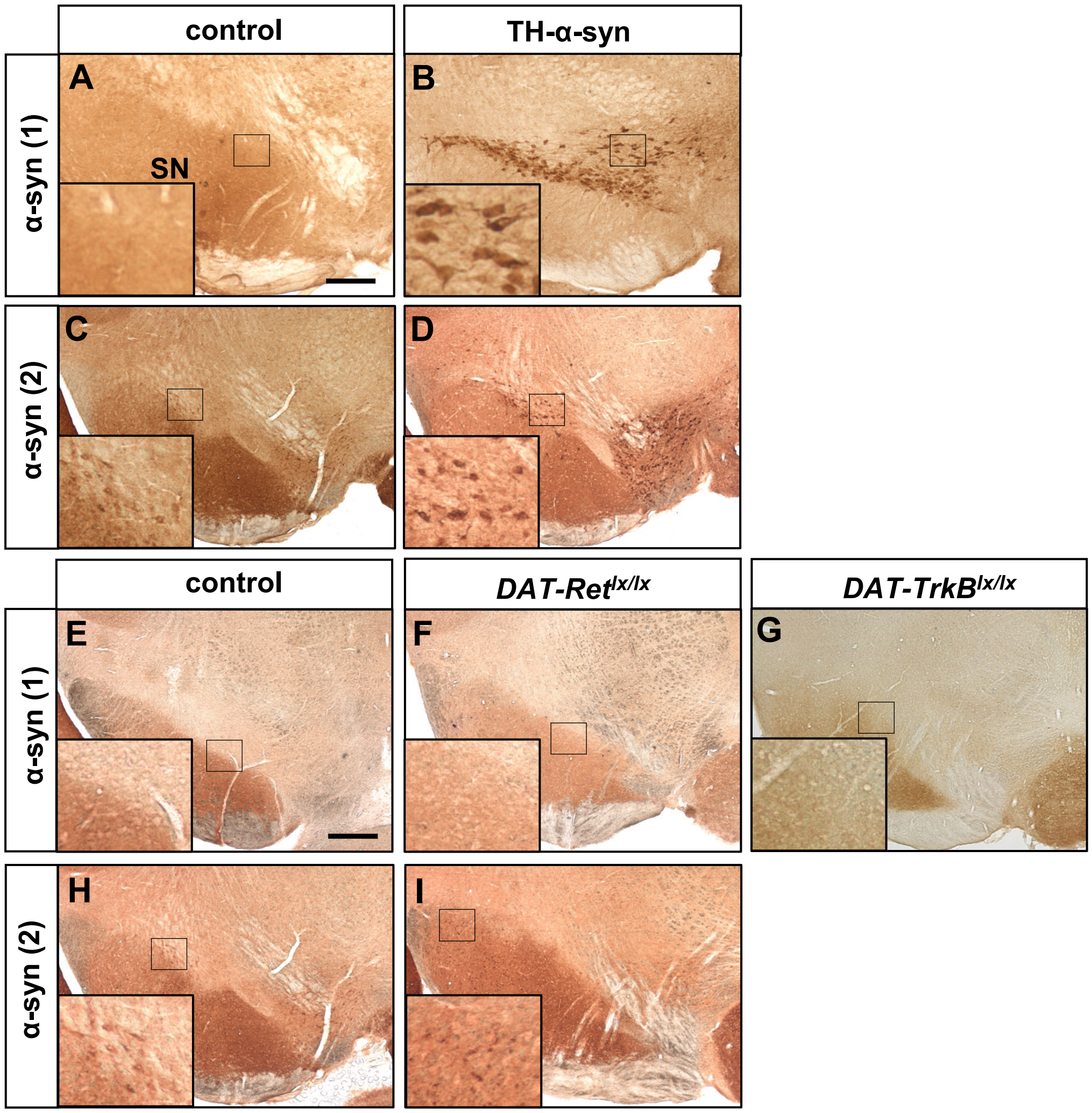
control
TH-α-syn
α-syn (1)
A
SN
B
α-syn (2)
C
D
control
DAT-Ret^lx/lx
DAT-TrkB^lx/lx
α-syn (1)
E
F
G
α-syn (2)
H
I
