# Supplementary material for: Absence of Ret Signaling in Mice Causes Progressive and Late Degeneration of the Nigrostriatal System
Source: PLoS Biol. 2007 Feb 13;5(3):e39. doi: 10.1371/journal.pbio.0050039 (PMC1808500; doi:10.1371/journal.pbio.0050039)
Supplement: Supplementary file 4 [file pbio.0050039.sg004.pdf]

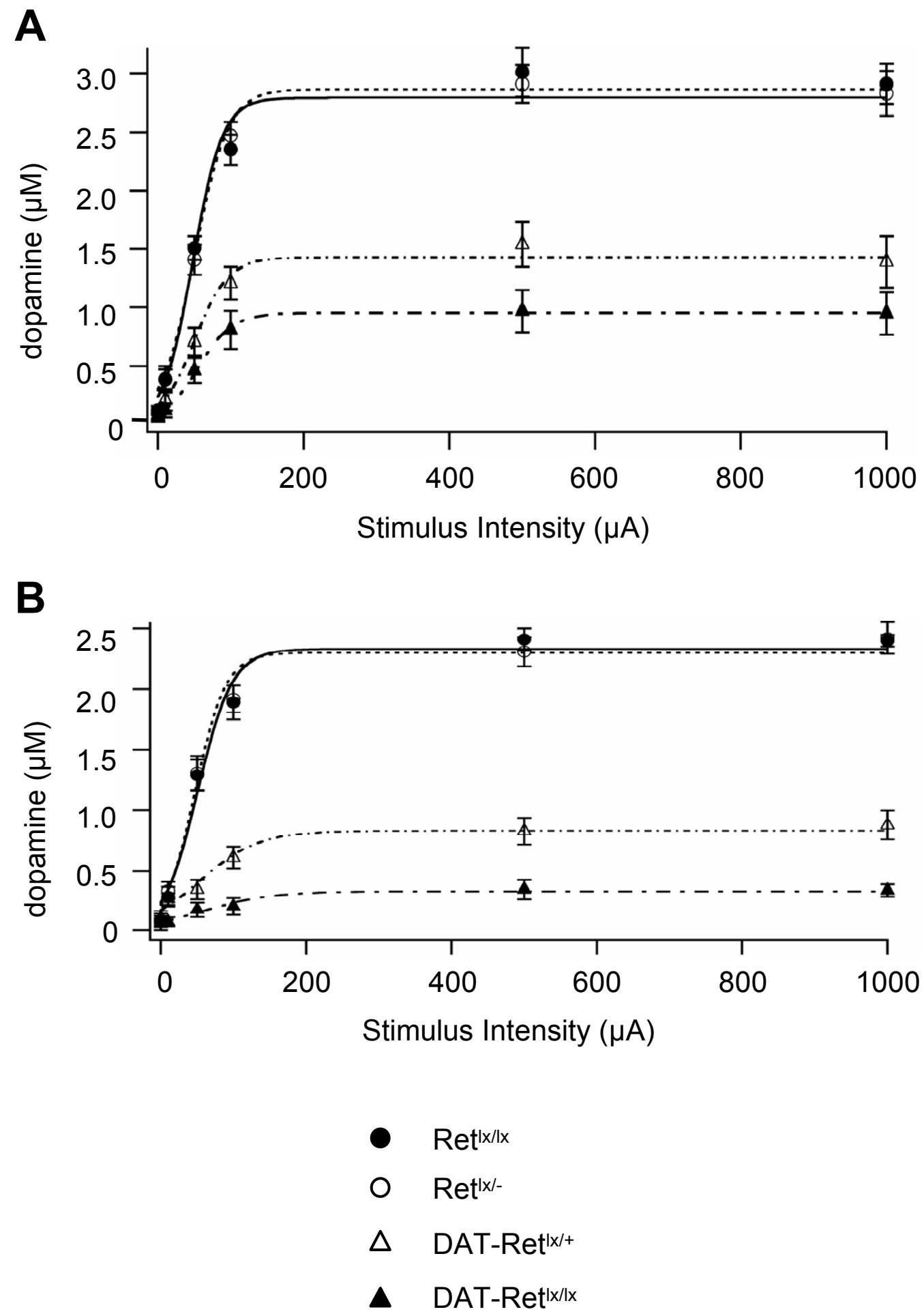
A
dopamine (µM)
3.0
2.5
2.0
1.5
1.0
0.5
0
0
200
400
600
800
1000
Stimulus Intensity (µA)
B
dopamine (µM)
2.5
2.0
1.5
1.0
0.5
0
0
200
400
600
800
1000
Stimulus Intensity (µA)
Ret lx/lx
Ret lx/-
DAT-Ret lx/+
DAT-Ret lx/lx
