# Supplementary material for: Absence of Ret Signaling in Mice Causes Progressive and Late Degeneration of the Nigrostriatal System
Source: PLoS Biol. 2007 Feb 13;5(3):e39. doi: 10.1371/journal.pbio.0050039 (PMC1808500; doi:10.1371/journal.pbio.0050039)
Supplement: Supplementary file 5 [file pbio.0050039.sg005.pdf]

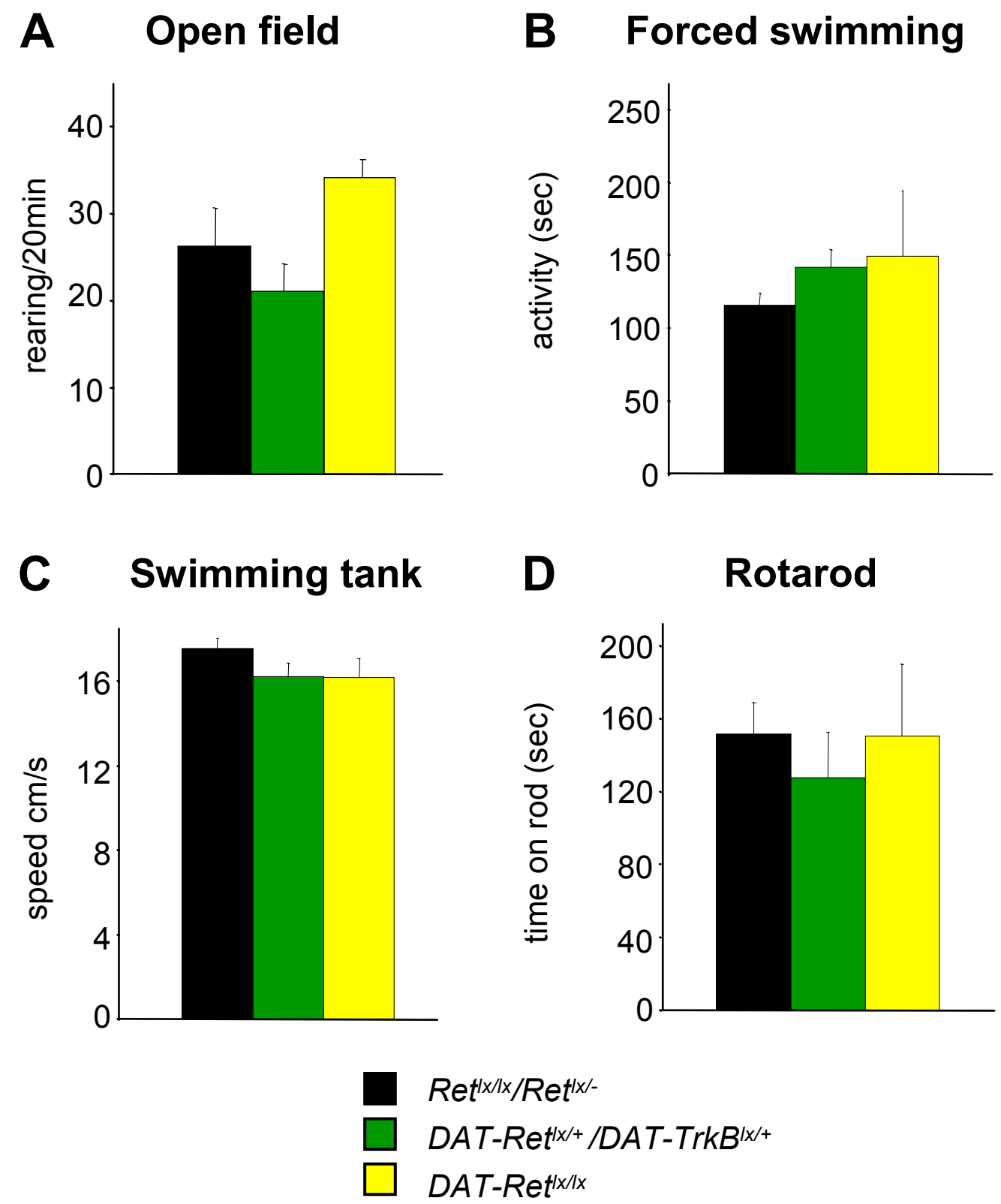

A
Open field
rearing/20min
0
10
20
30
40
B
Forced swimming
activity (sec)
0
50
100
150
200
250
C
Swimming tank
speed cm/s
0
4
8
12
16
D
Rotarod
time on rod (sec)
0
40
80
120
160
200
Ret lx/lx/Ret lx/-
DAT-Ret lx/+ /DAT-TrkB lx/+
DAT-Ret lx/lx
