# Supplementary material for: Absence of Ret Signaling in Mice Causes Progressive and Late Degeneration of the Nigrostriatal System
Source: PLoS Biol. 2007 Feb 13;5(3):e39. doi: 10.1371/journal.pbio.0050039 (PMC1808500; doi:10.1371/journal.pbio.0050039)
Supplement: Supplementary file 6 [file pbio.0050039.sd001.doc]

**Supplementary Materials and Methods**

**Immunohistochemistry**

Tissue was prepared as described above and primary antibodies used were purified sheep anti-alpha synuclein (1:500, Chemicon), and a rabbit anti-alpha synuclein crude serum (1:1000, Chemicon) [1]. Nissl staining was performed according to standard procedures.

**Single Cell RT-PCR**

For single cell harvesting unfixed brains from 3 month old mice of the indicated genotypes were cut with a cryostat in 10 µm thick coronal sections and collected on slides covered by a PEN film (1.35 µm; PALM microlaser, Bernried, Germany) and stored at -80 ºC. Slides were briefly dried before use and single cells were collected with a PALM microdissection setup (PALM microlaser, Bernried, Germany). For reverse transcription and first round of PCR amplification a one-step RT-PCR kit (Qiagen, Hilden, Germany) was used according to the provider’s instructions, followed by a nested second PCR reaction. For both PCR reactions specific primers for TrkB [2] were used together with specific primers for TH and Girk2 as markers for dopaminergic neurons, GAD67 and GAD65 as markers for GABAergic neurons, and GFAP as a marker for astroglia [3]. The cycling conditions for the RT-PCR reaction were 30 min at 50 ºC, 15 min at 94 ºC, 35 cycles of 30 sec at 94 ºC, 1 min at 55 ºC and 2 min at 72 ºC, followed by 10 min at 72 ºC. For the nested PCR we used 3 min at 94 ºC , 35 cycles of 30 sec at 94 ºC , 30 sec at 58 ºC , and 1 min at 72 ºC , followed by 10 min at 72 ºC. The nested PCR was performed individually for each primer pair in 50 µl using 2.5 µl of the first PCR product, 1 µM of each primer and 4 units of Taq polymerase. We confirmed the results of the nested TrkB PCR (primers surrounding the floxed exon K2) by two independent PCR primer sets, one set with a primer 5’ to the floxed exon (primer A = AGTTGGCGAGACATTCCAAG) and a second primer within the floxed exon (primer B = CCCTAAGGAACTTGTTGAGG) and one set with both primers within the floxed exon (primer C = GCACGCAAGGACTTTCATCG; primer B) The PCR products were separated and visualized in an ethidium bromide-stained agarose gel (2%). From the 200-250 isolated cells of the substantia nigra of each genotype, around half of these cells were positive by RT-PCR for TH. In the tissue from wild type mice we were able to detect TrkB in 63 cells out of 130 TH positive cells. In the DAT-TrkB lx/lx mutant mice only 19 out of 109 TH positive cells were also positive for TrkB.

**Behavioral tests**

Experimental protocols were approved by the government of Oberbayern, Germany. 24-months old mice were housed individually with free access to water and food in a room with 12h/12h reversed day-night cycle. All experiments were conducted during the night period in a quiet room by 12 lux light.

Open field:

Mice were placed into a 59cm x 59cm large arena for 10 min and their horizontal and vertical movement monitored by an observer and by using EthoVision (Noldus, Sterling, USA). The experiment was repeated on the consecutive day and the average of the two trails per mouse was taken for further analysis [4].

Swimming tank:

To monitor leg movements, mice were swimming 60 cm in a water filled glass-tank (100cm long and 6 cm wide, filled with 23ºC wate to a depth of 20 cm) to a visible black escape platform (6cm long, 20.5 cm high) 0.5 cm above the water level [5]. Mice were given three training sessions per day for three consecutive days in which they were allowed to swim 4 times per session. During the tests, mice were tested 6 times per day (in two separate test sessions each comprising 3 consecutive runs) during two consecutive days. The tests were videotaped and the time measured till they reached the platform.

Forced swimming test:

The test was performed as described previously [6]. Briefly, to test spontaneous activity, mice (without any previous training) were placed in a plexiglas cylinder (20 cm high and 14 cm in diameter) filled with water to a height of 10cm. A camera was used to observe and record the animal’s behavior. During the 6 minutes of the trial, the first 2 min the animal was allowed to adjust to the new conditions. In the next 4 min, the duration of the two types of motor activity, struggling and floating status, was recorded. The results are expressed as activity (sec)/240sec.

Rotarod:

To monitor limbs motor coordination and balance, a computerized rotarod apparatus was used (TSE, Bad Homburg, Germany). Mice were trained at several constant speeds (0, 4, 10, 25 rpm) and increasing speeds (4-10, 10-20, 20-30 rpm) three times five minutes each and were placed back on the rod every time they fell. After the mice attained a steady baseline level of performance, they were tested 4 times five minutes at 25 rpm (in separate trials, during one day) and 4 times five minutes at increasing speed 4-25 rpm (separate trials, the next day). The latency to fall off the rotarod during the 300 sec test was recorded [4].

**Supplementary references:**

1. von Bohlen und Halbach O, Minichiello L, Unsicker K (2005) Haploin-sufficiency for trkB and trkC receptors induces cell loss and accumulation of alpha-synuclein in the substantia nigra. Faseb J 19: 1740-1742.

2. Gonzalez M, Ruggiero FP, Chang Q, Shi YJ, Rich MM, et al. (1999) Disruption of Trkb-mediated signaling induces disassembly of postsynaptic receptor clusters at neuromuscular junctions. Neuron 24: 567-583.

3. Liss B, Bruns R, Roeper J (1999) Alternative sulfonylurea receptor expression defines metabolic sensitivity of K-ATP channels in dopaminergic midbrain neurons. Embo J 18: 833-846.

4. Valenti P, Cozzio A, Nishida N, Wolfer DP, Sakaguchi S, et al. (2001) Similar target, different effects: late-onset ataxia and spatial learning in prion protein-deficient mouse lines. Neurogenetics 3: 173-184.

5. Carter RJ, Lione LA, Humby T, Mangiarini L, Mahal A, et al. (1999) Characterization of progressive motor deficits in mice transgenic for the human Huntington's disease mutation. J Neurosci 19: 3248-3257.

6. Bilkei-Gorzo A, Racz I, Michel K, Zimmer A (2002) Diminished anxiety- and depression-related behaviors in mice with selective deletion of the Tac1 gene. J Neurosci 22: 10046-10052.
